# Supplementary material for: Unraveling the impact of hyperleptinemia on female reproduction: insights from transgenic pig model
Source: Biol Res. 2024 Sep 4;57:60. doi: 10.1186/s40659-024-00545-7 (PMC11373500; doi:10.1186/s40659-024-00545-7)
Supplement: Supplementary file 1 — Supplementary Material 1 [file 40659_2024_545_MOESM1_ESM.docx]

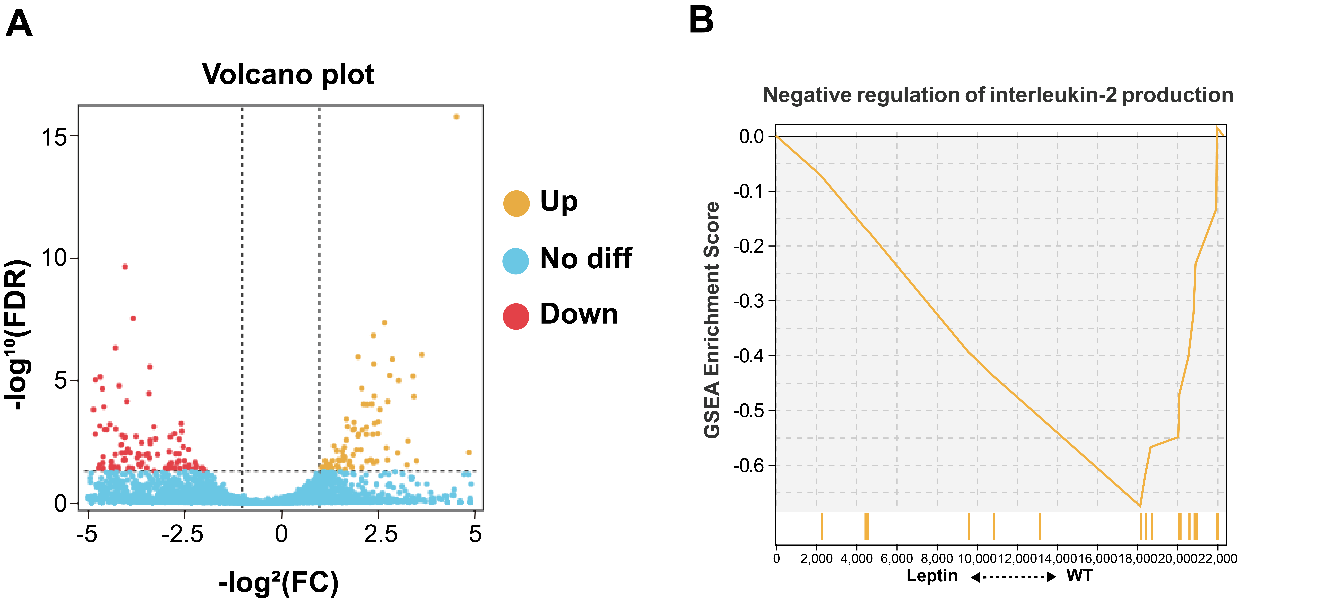


**Fig. S1. (A)** Volcano plots of DEGs in leptin and WT group **(B)** The GSEA result of ovarian inflammation.


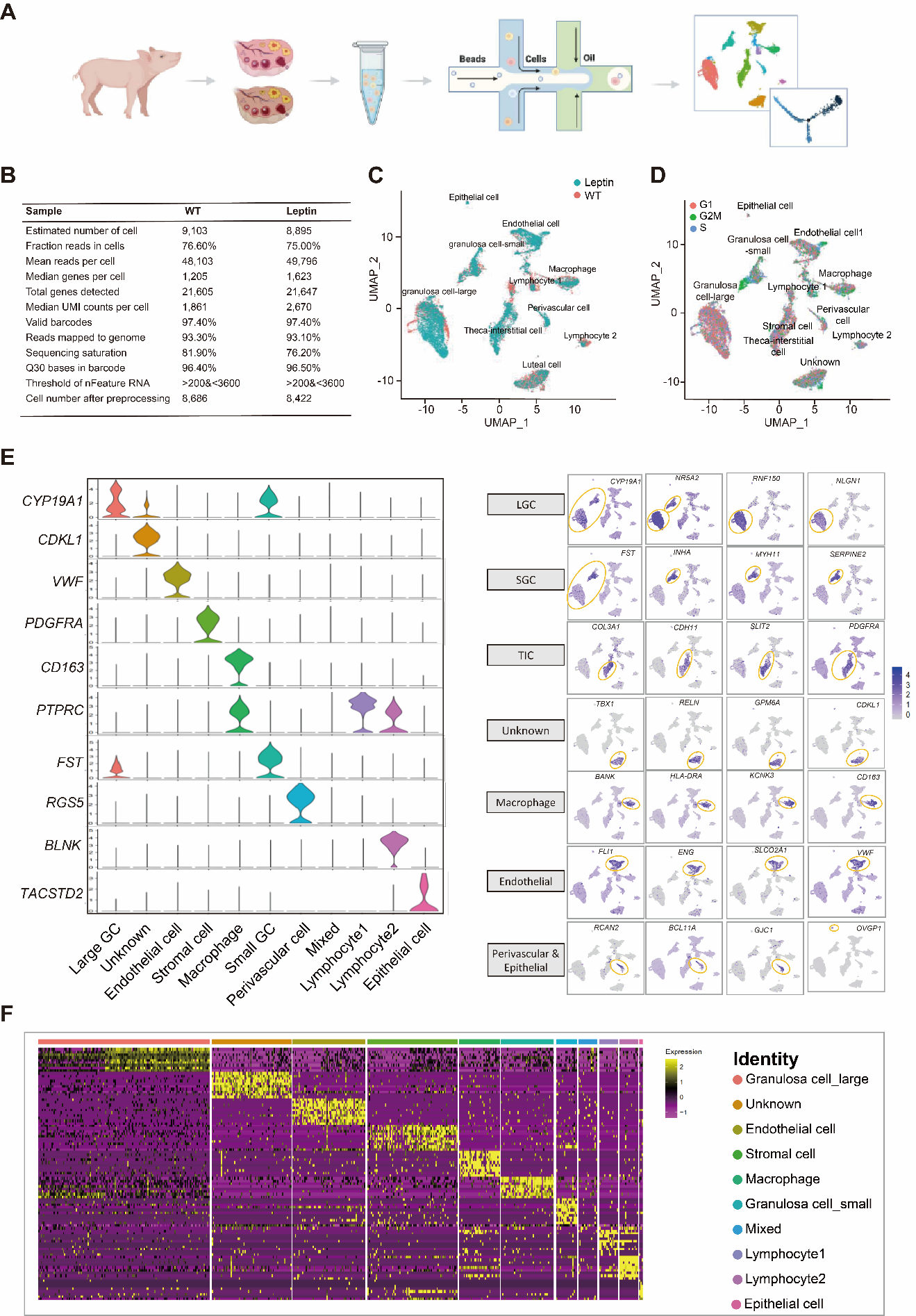


**Fig. S2: Sequencing information and novel marker genes. (A)** Schematic diagram of snRNA sequencing. **(B)** Summary information of sample data identified by CellRanger. **(C)** UMAP plot showing sample identity (WT and leptin) **(D)** UMAP clustering of 3 phases in different cell populations. **(E)** Distribution of feature genes at different cell clusters. The gene expression levels are indicated by the colors of the bar. **(F)** Heatmap expression of upregulated genes in different cell populations.

**
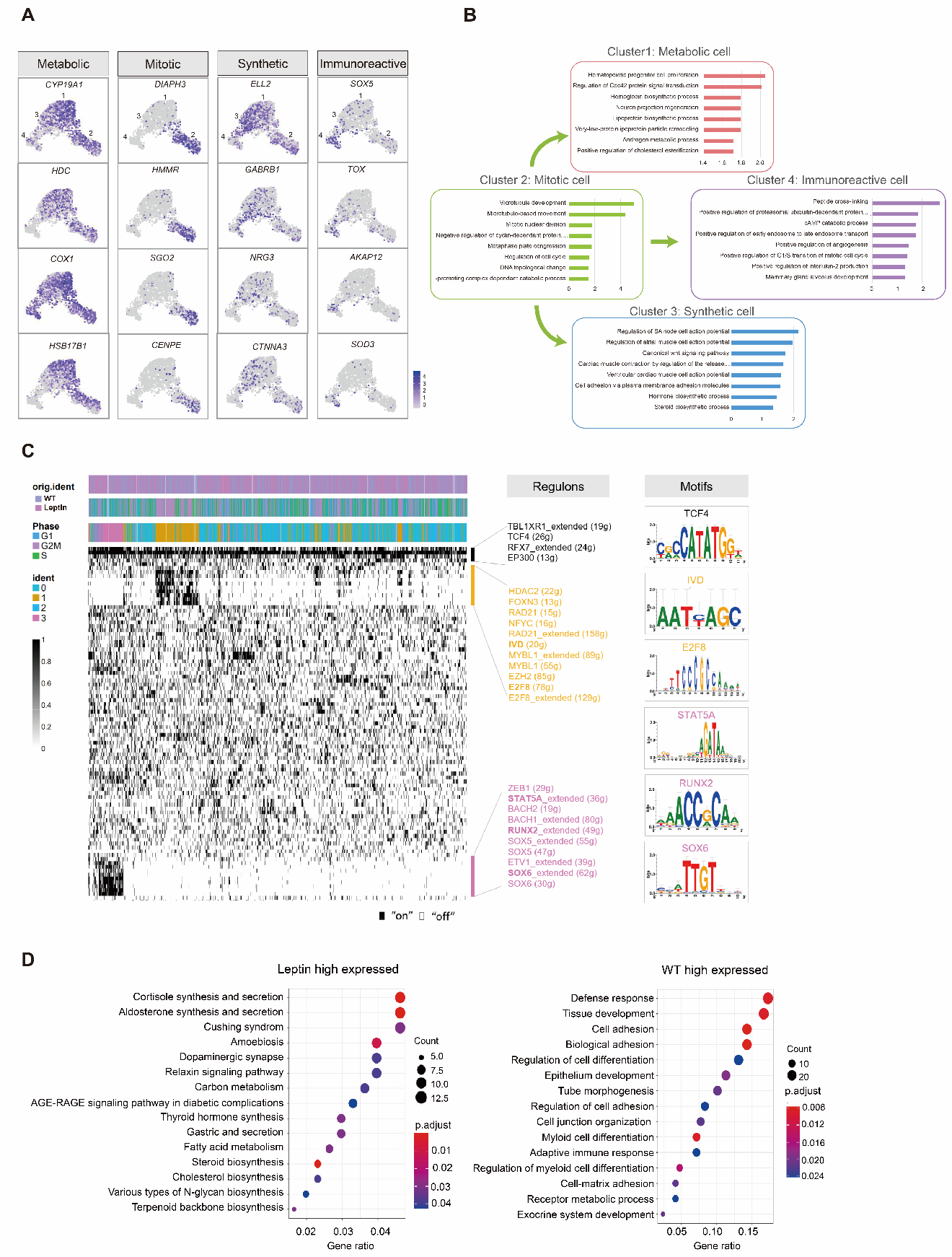
**

**Fig. S3: Gene expression pattern of ovarian cell subtypes (A)** Distribution of feature genes at four different clusters of SGC. The gene expression levels are indicated by the colors of the bar. **(B)** Representative GO terms of highly expressed genes in leptin and WT pig ovaries. **(C)** Mapping regulons activity of transcriptional factors in SGCs using SCENIC. Cluster labels correspond to 4 subtypes in Fig. 4.D and cell state; TFs confirmed by literature (Regulons) and their corresponding enriched DNA-binding motifs which are shown as (Motifs). **(D)** Representative KEGG terms of highly expressed genes in leptin group (left) and GO Biological Process terms of highly expressed genes in WT group (right).

.

| **Table S1: List of primers for PCR** | | | | |
| --- | --- | --- | --- | --- |
| Gene symbol | Gene ID. | Product size | Primer | Sequence (5’-3’) |
| pleptin | XM_021078503 | 208 | Forward | CTTTGGCCCTATCTGTCCTACGTTG |
|  |  |  | Reverse | TCGCCAGGGTCTGGTCCATCT |
| pβ-actin | AY550069 | 142 | Forward | ATCAGCAAGCAGGAGTACGACG |
|  |  |  | Reverse | GCCATGCCAATCTCATCTCATTTT |
| GFP* |  | 136 | Forward | ACCCTCGTGACCACCCTGACCT |
|  |  |  | Reverse | TCTTGTAGTTGCCGTCGTCCTT |
| *This sequences originated from pIRES2-AcGFP1( https://www.addgene.org/browse/sequence_vdb/6217/) | | | | |

| **Table S2: Animal information** | | | | | |
| --- | --- | --- | --- | --- | --- |
| Experiment title | Number of pigs | Pig Id | Weight (kg) | Age (days) | Comments |
| Body growth | Leptin pig n=5 | LepF2-02, LepF2-04, LepF2-10, LepF2-12, LepF2-18, | 21-31.5 | 150 | Presented in Figure 1 |
|  | WT n=4 | H400, H401, H427, H467 | 95-112 | 150 |  |
| Reproductive performance | Leptin pig n=5 | LepF2-02, LepF2-04, LepF2-12, LepF2-18, LepF2-33 | 21-31.5 | 150 | Varied with age and is presented in Figure 2 |
|  | WT n=4 | H400, H401, H427, H467 | 95-112 | 120-130 |  |
| Serum hormones and fatty acids | Leptin pig n=4 | LepF2-02, LepF2-04, LepF2-12, LepF2-18 | 80-151 kg | 330-450 |  |
|  | WT n=4 | H400, H401, H427, H467 | 138-163 kg | 300 |  |
| Protein analysis, and Sequencing | Leptin pig n=3 | LepF3-18, LepF3-21, LepF3-23 | 50-100 | 430-440 | 1^st^ estrus at 290 days, 355 days, and No estrus till sampling |
|  | WT n=3 | P017, P030, P070 | 186-200 | 440 | 1^st^ estrus at 140-150 days |

| **Table S3: List of primers for qPCR** | | |
| --- | --- | --- |
| Gene symbol | Primer Sequence (5’-3’) | |
| pleptin | F: GGCCCCAGAAGCACATCC | R:TCAGCAGCCAGGGCTGAG |
| StAR | F: TGGGGCCCCGAGACTTTGTGA | R: GTCTTCGGCAGCCATCCCTTGAG |
| CYP11A1 | F: CGCCAGGCCCAAGGAGACACAA | R: GCCCCAGCCAAAGCCCAAGTTC |
| CYP17A1 | F: TGGTAAAGTGGATCGTGGCC | R: TCGCCAATGCTGGAGTCAAT |
| CYP19A1 | F: ATGCATGGCAAGCTCTCCTT | R: CGCCACGTTTCTCAGCAAAA |
| ESR1 | F: GGCCAAGCCCTCTCTTGATT | R: AGGATCTCTAGCCAGGCACA |
| FSHR | F: CCAGGAGAGCAAGGTGACAG | R: GGAGGTTGGGAAGGTTCTGG |
| LHCGR | F: GATGCACAATGACGCCTTCC | R: TGGTCTCCTTGCTGTGCTTT |
| GAPDH | F: ACACTCACTCTTCTACCTTTG | R: CAAATTCATTGTCGTACCAG |

| **Table S4: List of proteins** | | | | |
| --- | --- | --- | --- | --- |
| **Protein Name** | **Cas#** | **Company** | **Source** | **Dose** |
| anti-β-actin (β-actin) | A5441 | Sigma-Aldrich | Mouse | 1:2000 |
| FSHR Polyclonal antibody | 22665-1-AP | Proteintech | Rabbit | 1:2000 |
| LHCGR Polyclonal antibody | 19968-1-AP |  |  | 1:1000 |
| StAR Polyclonal antibody | 12225-1-AP |  |  | 1:1000 |
| Cytochrome P450 17A1 Antibody (CYP17A1) | 14447-1-AP |  |  | 1:1000 |
| Cytochrome P450 19A1 Antibody (CYP19A1) | AF5229 | Affinity | Rabbit | 1:2000 |
| ERα (F-10) (ESR1) | SC-8002 | Santa Cruz Biotechnology, Inc | Mouse | 1:1000 |
| Caspase-3 Antibody | #9662 | Cell Signaling Technology | Rabbit | 1:1000 |
